# Model-informed approach for risk management of bleeding toxicities for bintrafusp alfa, a bifunctional fusion protein targeting TGF-β and PD-L1

**DOI:** 10.1007/s00280-022-04468-6

**Published:** 2022-09-06

**Authors:** Yulia Vugmeyster, Ana-Marija Grisic, Justin J. Wilkins, Anja H. Loos, Roland Hallwachs, Motonobu Osada, Karthik Venkatakrishnan, Akash Khandelwal

**Affiliations:** 1 EMD Serono Research and Development Institute, Inc., An Affiliate of Merck KGaA, 45 Middlesex Turnpike, Billerica, MA 01821 USA; 2 Merck Healthcare KGaA, Frankfurter Str. 250, 64293 Darmstadt, Germany; 3 Occams, Amstelveen, The Netherlands; 4 Merck Biopharma Co., Ltd., An Affiliate of Merck KGaA, Tokyo, Japan

**Keywords:** Clinical pharmacokinetics, Exposure–response relationship, Immune checkpoint inhibitor, Phase 1, 2, 3 trials, Solid tumors

## Abstract

**Purpose:**

Bintrafusp alfa (BA) is a bifunctional fusion protein composed of the extracellular domain of the transforming growth factor-β (TGF-β) receptor II fused to a human immunoglobulin G1 antibody blocking programmed death ligand 1 (PD-L1). The recommended phase 2 dose (RP2D) was selected based on phase 1 efficacy, safety, and pharmacokinetic (PK)–pharmacodynamic data, assuming continuous inhibition of PD-L1 and TGF-β is required. Here, we describe a model-informed dose modification approach for risk management of BA-associated bleeding adverse events (AEs).

**Methods:**

The PK and AE data from studies NCT02517398, NCT02699515, NCT03840915, and NCT04246489 (*n* = 936) were used. Logistic regression analyses were conducted to evaluate potential relationships between bleeding AEs and BA time-averaged concentration (*C*_avg_), derived using a population PK model. The percentage of patients with trough concentrations associated with PD-L1 or TGF-β inhibition across various dosing regimens was derived.

**Results:**

The probability of bleeding AEs increased with increasing *C*_avg_; 50% dose reduction was chosen based on the integration of modeling and clinical considerations. The resulting AE management guidance to investigators regarding temporary or permanent treatment discontinuation was further refined with recommendations on restarting at RP2D or at 50% dose, depending on the grade and type of bleeding (tumoral versus nontumoral) and investigator assessment of risk of additional bleeding.

**Conclusion:**

A pragmatic model-informed approach for management of bleeding AEs was implemented in ongoing clinical trials of BA. This approach is expected to improve benefit-risk profile; however, its effectiveness will need to be evaluated based on safety data generated after implementation.

**Supplementary Information:**

The online version contains supplementary material available at 10.1007/s00280-022-04468-6.

## Introduction

Bintrafusp alfa (BA, also referred to as MSB0011359C [M7824]) is a first-in-class bifunctional fusion protein composed of the extracellular domain of the transforming growth factor-β (TGF-β) receptor II (a TGF-β "trap") fused to a human immunoglobulin G1 antibody blocking programmed death ligand 1 (PD-L1) [1]. BA is thus designed to target tumors via colocalized, simultaneous inhibition of two key immunosuppression pathways in the tumor microenvironment: the PD-L1/programmed cell death 1 protein immune checkpoint pathway (targeting PD-L1) and immunosuppressive axis of TGF-β during tumor pathogenesis [2]. There are three identified isoforms of TGF-β (TGF-β1, -β2, and -β3), which are secreted as inactive polypeptides and bind to TGF-β receptors [3]. BA binds to all three isoforms, although with different affinity, with lowest affinity towards TGF-β2, due to low intrinsic binding affinity between TGF-β2 and TGF-β receptor [4].

The recommended phase 2 dose (RP2D) of 1200 mg every 2 weeks (Q2W) or 2400 mg every 3 weeks (Q3W) was selected based on integration of pharmacokinetic (PK)–pharmacodynamic (PD) data and exposure–response modeling, simulations, and all available data from single-agent phase 1 studies [5]. Specifically, a dose of 1200 mg Q2W was predicted to maintain serum trough concentrations (*C*_trough_) that inhibit all four targets of BA (PD-L1, and TGF-β1, -β2, and -β3) in circulation in > 95% of patients, and a dose of 2400 mg Q3W was predicted to have similar *C*_trough_. BA pharmacokinetics were shown to be unaffected by concomitant chemotherapies [6], supporting the selection of a BA dose of 2400 mg Q3W for combination with chemotherapies that are administered on a Q3W cycle.

In multiple expansion cohorts of phase 1 trials NCT02517398 and NCT02699515, BA has demonstrated antitumor activity and a manageable safety profile when administered as a single agent at a dose of 1200 mg Q2W [1, 7–11]. In addition, BA is being investigated in combination with chemotherapies or targeted agents in several tumor types (e.g., non-small cell lung cancer [NSCLC], cervical cancer [CC], and biliary tract cancer [BTC]) at a dose of either 2400 mg Q3W or 1200 mg Q2W (NCT03840902, NCT0455195, and NCT04066491), and the safety profile of BA with combination regimens is also considered manageable based on the data available to date [12].

BA-associated adverse events (AEs) of special interest included immune-related AEs (irAEs), anemia, bleeding events, infusion-related reactions, and TGF-β inhibition-mediated skin AEs and were mostly mild to moderate [13]. Among the above listed AEs of special interest, bleeding and TGF-β inhibition-mediated skin AEs are thought to be associated with TGF-β inhibition [13–15], whereas irAEs are associated with PD-L1 blockade as seen for other immune checkpoint inhibitors interfering with the PD-1/PD-L1 axis [16, 17]. Specifically, for bleeding, it has been reported that treatment with anti-TGF-β neutralizing monoclonal antibody (blocking all three isoforms) was associated with an increased risk of bleeding and cardiac toxicity in mice and monkeys [18]. Cardiovascular toxicities and systemic bleeding were also reported for small molecule TGF-β receptor antagonists [19]; however, cardiac toxicity was not observed in the clinical studies of BA.

The observed incidence of bleeding AEs in patients treated with BA in phase 1 studies was 39.3% for all grades and 10.2% for grade 3 or higher [13]. The most common bleeding AEs (overall incidence > 5%) with BA treatment were grade 1 or 2 (mild or moderate) epistaxis, hemoptysis, gingival bleeding, hematuria, and rectal hemorrhage. The most frequent bleeding events of grade 3 or higher (severe) were gastrointestinal (GI) hemorrhage (1.3%) and tumor hemorrhage (1.7%) [13]. The first onset of bleeding events was typically early, within 12 weeks of treatment. Initial observations in early clinical development of BA suggested a higher frequency of bleeding AEs as seen in BA compared with other immune checkpoint inhibitors [20] or targeted agents [21]. Accordingly, risk mitigation measures (such as specific exclusion criteria related to history of bleeding, treatment interruption and discontinuation) had already been implemented in ongoing phase 1 and 2 BA studies throughout the program. Of note, as is typical for currently approved immuno-oncology biotherapeutics (i.e., the immune checkpoint inhibitors), treatment interruptions rather than dose reductions or modifications were recommended for relevant treatment-emergent toxicities, including bleeding. The preliminary results from ongoing BA/chemotherapy combination studies with 2400 mg Q3W dosing suggested that bleeding frequency was higher than previously observed in monotherapy studies, especially in GI. Thus, additional risk mitigation measures were evaluated based on pharmacological considerations of the dual mechanism of action, with the intent to keep patients with higher grade bleedings on BA treatment by reducing the risk for bleeding recurrences and optimizing BA exposure for these patients. Specifically, in this report, we describe a dose modification approach for BA-associated bleeding AE management, informed by population PK modeling, exposure-safety modeling, PK-PD data, and current understanding of the underlying AE mechanisms. To our knowledge, this is the first example of model-informed dose modification for management of AEs for a bifunctional therapeutic protein in immuno-oncology.

## Methods

Study designs of each study were previously described [9, 11, 12] and are summarized in Table 1.

**Table 1:** Studies and endpoints included in exposure-safety analysis

NCT number and reference for study results	NCT02517398 [9]	NCT02699515 [11]	NCT03840915 [12]	NCT04246489 [30]	Total
Phase	1	1	1	2	–
Tumor type	DE: solid tumors Exp cohorts: various tumor types^a^		NSCLC	BTC	–
Combination agent	–	–	Chemotherapies	–	–
*N*	593	114	70	159	936
Dose level					
1 mg/kg Q2W, *n*	3	–	–	–	3
3 mg/kg Q2W, *n*	9	7	–	–	16
10 mg/kg Q2W, *n*	6	9	–	–	15
20 mg/kg Q2W, *n*	10	7	–	–	17
30 mg/kg Q2W, *n*	7	–	–	–	7
500 mg Q2W, *n*	40	–	–	–	40
1200 mg Q2W, *n*	515	91	–	159	765
2400 mg Q2W, *n*	3	–	–	–	3
2400 mg Q3W, *n*	–	–	70	–	70
Bleed_TEAE incidence, %	39.3	29.8	55.7	17.6	35.7
Bleed_TEAE3 incidence, %	9.61	10.5	10	2.52	8.55
Bleed_GI incidence, %	16.0	14.0	22.9	11.3	15.5
Bleed_GI3 incidence, %	4.38	6.14	7.14	2.52	4.49

*BTC* biliary tract cancer, *DE* dose escalation, *Exp* expansion, *NSCLC* non-small cell lung cancer, *Q2W* every 2 weeks, *Q3W* every 3 weeks, bleed_TEAE, treatment-related bleeding events of any grade, *bleed_TEAE3* treatment-related bleeding events of grade ≥ 3, *bleed_GI* gastrointestinal bleeding events of any grade, *bleed_GI3* gastrointestinal bleeding events of grade ≥ 3

^a^See Supplemental Table 1 for additional details

### Population PK and exposure derivation

The population PK (popPK) model was developed based on data from 873 patients (9792 observations) from clinical trials NCT02517398, NCT02699515, and NCT04246489 who received various doses of BA (shown in Table 1), using the nonlinear mixed-effects modeling approach. Covariate model development comprised a full covariate modeling approach and a subsequent model reduction step via backwards elimination (*p* < 0.05, based on the log-likelihood ratio test), as described in Supplemental Methods. The final model (shown in Supplemental Table 1, with goodness-of-fit plots and a visual predictive check shown in Supplemental Figs. 1 and 2, respectively) was a two-compartment model with time-varying linear clearance (CL), baseline body weight effect on CL, central volume, peripheral volume, and intercompartmental clearance, and the following additional baseline covariate effects: antidrug antibody status (ever/never positive), albumin concentration, C-reactive protein concentration, estimated glomerular filtration rate, international normalized ratio, platelet count, sex, tumor size, tumor type, and white blood cell count on CL, and albumin concentration, international normalized ratio, platelet count, sex, tumor size, and white blood cell count on central volume.

**Fig. 1:**
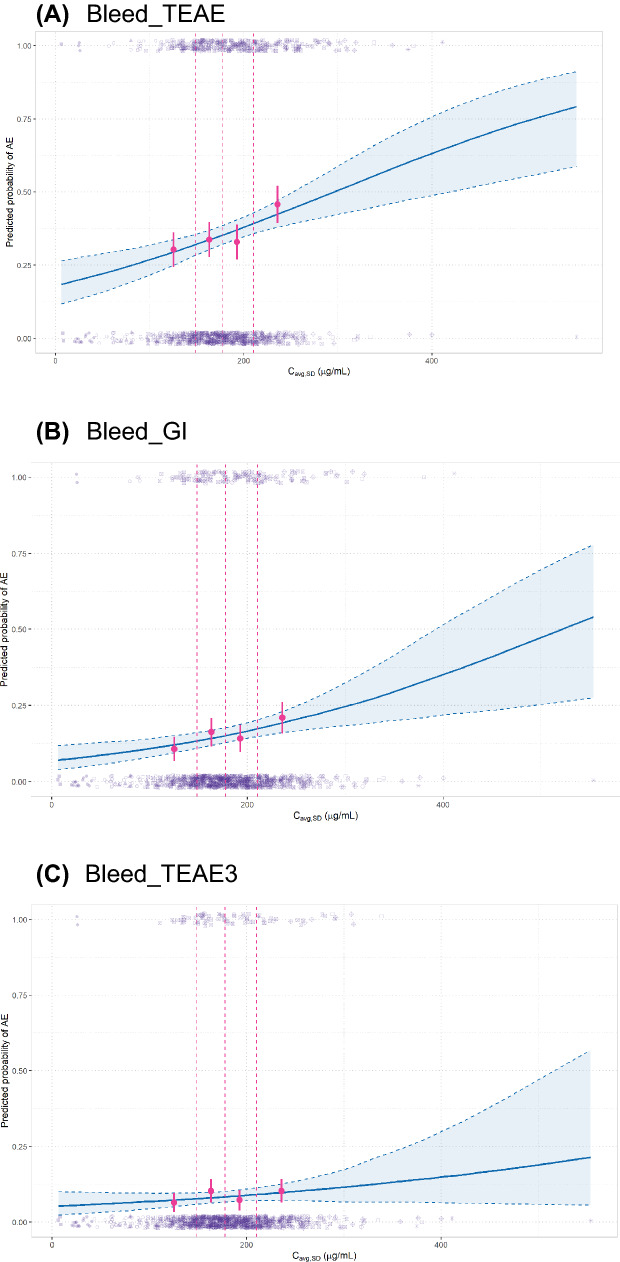
Univariable exposure-safety analysis for bleed_TEAE (**A**), bleed_GI (**B**), and bleed_TEAE3 (**C**). Blue line and shaded area represent model-predicted AE probability (median and 95% CI); pink circles represent observed AE incidence by quartiles of exposure and are placed at 12.5th, 37.5th, 62.5th, and 87.5th percentiles of the exposure distribution (i.e., median for each exposure quartile); the error bars represent 95% CIs; pink dotted lines represent boundaries of exposure quartiles; purple dots individual patient data. *C*_avg,SD_, average bintrafusp alfa concentration over the dosing interval, *bleed_TEAE* treatment-related bleeding events of any grade, *bleed_TEAE3* treatment-related bleeding events of grade ≥ 3, *bleed_GI* gastrointestinal bleeding events of any grade;* CI* confidence interval

**Fig. 2:**
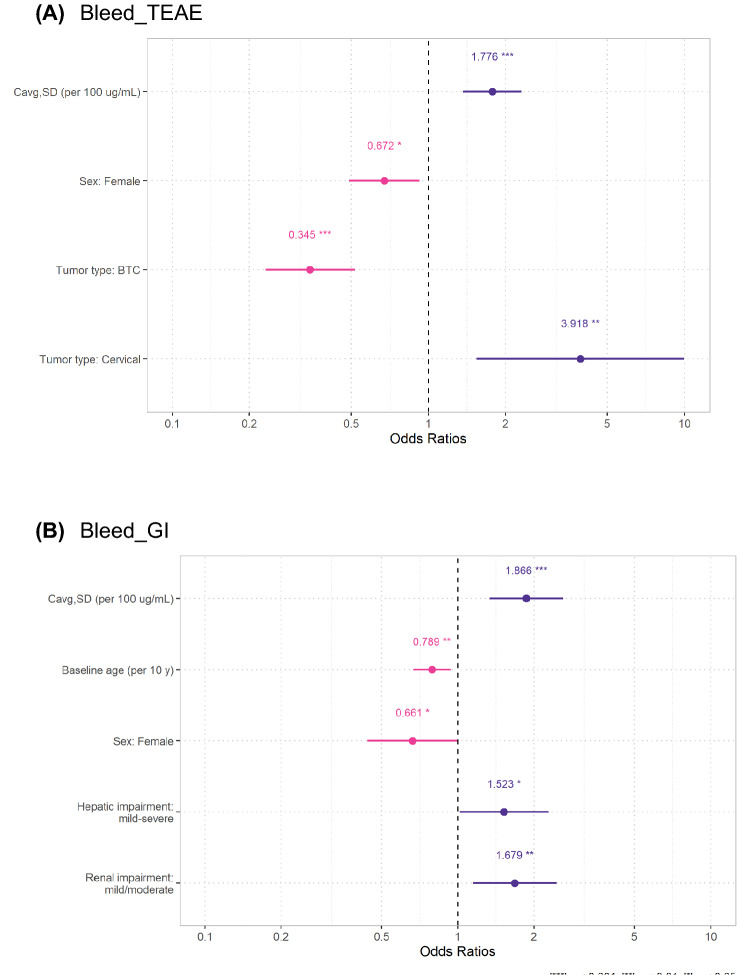
Final exposure-safety models for bleed_TEAE (**A**) and bleed_GI (**B**). Numbers are odds ratios; horizontal bars represent 95% CIs provided by reduced models. *C*_avg,SD_ average bintrafusp alfa concentration over the dosing interval, *bleed_TEAE* treatment-related bleeding events of any grade, *bleed_GI* gastrointestinal bleeding events of any grade, *BTC* biliary tract cancer. ***p < 0.001; **p < 0.01; *p < 0.05

Individual patient average concentrations over the dosing interval (tau, which is 336 and 504 h for Q2W and Q3W dosing, respectively) after a single dose (*C*_avg,SD_, calculated as area under the curve/tau) for exposure-safety analyses were predicted using the popPK model. For studies NCT02517398, NCT02699515, and NCT04246489, the final popPK model (Supplemental Table 1) was used, while for study NCT03840915, the base popPK model (with body weight as the only covariate) was used for predicting *C*_avg,SD_. Seven patients in NCT02517398 received a lower dose (either 1 or 0.3 mg/kg) in cycle 1 and were then switched to a pharmacologically active dose; these patients were excluded from exposure-safety analyses (but included in the popPK modeling). The use of BA exposure following the first dose was based on typical considerations for exposure–response analyses for biologics in oncology drug development, designed to minimize the multifactorial determinants of longitudinal changes in systemic exposure in the context of potential tumor response to treatment [22, 23].

Target *C*_trough_ levels associated with PD-L1 target occupancy and/or TGF-β neutralization were based on phase 1 PK-PD data [5]. Specifically, *C*_trough_ of ≥ 11 μg/mL was associated with PD-L1 target occupancy and TGF-β1 and TGF-β3 neutralization, while *C*_trough_ of ≥ 50 μg/mL was associated with TGF-β2 neutralization. The popPK simulated *C*_trough_ distributions were used to assess the percentage of patients who achieved these target *C*_trough_ levels across various dosing regimens.

### Definition of bleeding AEs and gastrointestinal bleeding AEs

The search list for bleeding AEs is based on the standardized MedDRA query (SMQ) ‘Haemorrhage terms (excl laboratory terms) (SMQ)’, version 23.0. This sub-SMQ is based on the SMQ ‘Haemorrhages (SMQ)’. Details on the selection of terms is provided in the ‘Introductory Guide for Standardised MedDRA Queries (SMQs) Version 23.0’ [24].

GI bleeding events were identified considering all preferred terms on the primary path to the system organ class gastrointestinal disorders within this SMQ. The limitation of preferred terms to their primary path was driven by the underlying clinical database and allowed a quick and pragmatic identification of GI-associated bleeding events.

### Exposure-safety modeling

The data for exposure-safety modeling included 936 patients receiving BA monotherapy (clinical trials NCT02517398, NCT02699515, and NCT04246489) and combination therapy (clinical trial NCT03840915) (Table 1 and Supplemental Table 2).

Outcomes of interest were treatment-emergent adverse events (TEAEs), observed any time after the start of treatment until 30 days after the last BA treatment, focusing on the following AEs:

- Treatment-emergent bleeding event of any grade (bleed_TEAE),
- Treatment-emergent bleeding event of grade ≥ 3 (bleed_TEAE3),
- GI bleeding event of any grade (bleed_GI),
- GI bleeding event of grade ≥ 3 (bleed_GI3).

Outcomes were classified as binary on patient level (AE observed, yes or no). Logistic regression analysis was used to investigate potential relationships between *C*_avg,SD_ and AEs of interest. R, version 4.0.5, was used to perform all analyses. First, the exposure-safety relationships were explored via univariable modeling; subsequently, other baseline predictors (Supplemental Tables 2 and 3) were assessed via full and stepwise-reduced (*p* < 0.05, based on log-likelihood ratio test) models if a significant univariable relationship with the exposure metric was identified. 100 μg/mL was chosen as the scaling unit for the odds ratio (OR), based on the median and range for *C*_avg_ in this dataset (Supplemental Fig. 1); this unit is approximately equal to the decrease in *C*_avg_ with a 50% dose reduction from 1200 mg QTW. Of note, four tumor type categories were evaluated (NSCLC, BTC, CC, and “other”), based on ongoing clinical trials in these indications at the time of the analysis; Asian versus non-Asian categories were also evaluated in exposure-safety models.

## Results

### Exposure-safety

The analyses included pooled dataset from global monotherapy phase 1 studies (dose escalation and 1200 mg Q2W expansion cohort), chemotherapy combination phase 1 study (2400 mg Q3W), and monotherapy phase 2 study (1200 mg Q2W) (Table 1). The observed incidence of various treatment-emergent bleeding AEs (such as bleed_TEAE, bleed_TEAE3, bleed_GI, and bleed_GI3 as defined in Methods) in this dataset by study is shown in Table 1. The observed incidence of any bleeding event at 1200 mg of BA Q2W in this dataset was 34.8% for any grade and 8.6% for grade ≥ 3.

Based on the univariable model, an association was identified between bleed_TEAE and *C*_avg,SD_ with an OR of 1.67 (95% CI, 1.3–2.14) per 100 μg/mL and between bleed_GI and *C*_avg,SD_ with an OR of 1.66 (95% CI, 1.21–2.27) per 100 μg/mL (Fig. 1A and B). As a sensitivity analysis, the univariable modeling was also performed on monotherapy data only (i.e., excluding NCT03840915) and similar exposure-safety associations for both bleed TEAE and bleed_GI were observed (Supplementary Fig. 3). In addition, the visual trend of increase in likelihood of bleeding with exposures was also observed in a chemotherapy combination dataset from NCT03840915 (26% bleed_GI at *C*_avg,SD_ ≥ median versus 20% bleed_GI at *C*_avg,SD_ < median), although the relatively small sample size (*n* = 70) precluded meaningful logistic regression modeling of these data. The pooling of NCT03840915 with the monotherapy data was considered justified, based on a similar trend of association between exposure and likelihood of bleeding compared with that seen with the monotherapy dataset. Exposure-safety associations were not discernable for bleed_TEAE3 or bleed_GI3, but a visual trend (not statistically significant) was observed for bleed_TEAE3 (Fig. 1C).

Covariate associations with prespecified demographic and disease-specific factors in addition to exposure were explored using a full and stepwise covariate modeling approach for bleed_TEAE and bleed_GI, as described in the Methods. The final models for bleed_TEAE and bleed_GI are shown in Fig. 2 and Supplemental Table 3. The BA exposure effect on probability of bleed_TEAE, bleed_GI, and magnitude and direction did not change after the addition of other predictors to the model compared with univariable exposure-AE models. For both bleed_TEAE and bleed_GI, several covariates were retained in the final model in addition to exposure. Notably, BTC tumor type was associated with lower probability of bleed TEAE, while CC tumor type was associated with higher probability of bleed TEAE. Tumor type was not associated with occurrence of bleed_GI. Increasing age, renal impairment, and hepatic impairment were associated with higher probability of bleed_GI, but not bleed_TEAE. Female sex was associated with higher probability of both bleed_GI and bleed_TEAE.

To evaluate potential benefit of dose modification for management of bleeding AEs, the univariable bleed_GI model was used to estimate the probability of bleed_GI and reduction in this probability for the considered dose modifications. The bleed_GI was chosen over bleed_TEAE, because the estimated ORs in the exposure-safety logistic regression analyses were similar for these two endpoints, but bleed_GI was deemed of higher clinical relevance and its incidence probability was independent of tumor type. The doses compared included 1200 mg Q2W, 2400 mg Q3W, 600 mg Q2W (given that BA was available in formulation units of 600 mg), and 1200 mg Q3W (based on clinical experience with Q2W and the available formulation strengths). The model-estimated probabilities of bleed_GI for various Q2W and Q3W doses of interest and model-predicted reduction in probability of bleed_GI are shown in Table 2. For example, based on these estimates, the probability of bleed_GI for a typical patient dosed with 1200 mg Q3W was reduced to ≈13% versus ≈22% with 2400 mg Q3W dosing. This relative decrease in probability of bleed_GI upon 50% dose reduction was deemed clinically meaningful.

**Table 2:** Probability of bleed_GI occurrence across dose levels of interest based on univariable model

Predicted *C*_avg,SD_	Probability (95% CI)
600 mg Q2W	
Geometric mean: 89 μg/mL	0.103 (0.074–0.137)
1200 mg Q2W	
Geometric mean: 178 μg/mL	0.151 (0.128–0.177)
1200 mg Q3W	
Geometric mean: 136 μg/mL	0.126 (0.101–0.155)
2400 mg Q3W	
Geometric mean: 273 μg/mL	0.222 (0.174–0.281)

*Bleed_GI *gastrointestinal (GI) bleeding event of any grade,* C*_*avg,SD*_ time-averaged concentration over the dosing interval after a single dose,* Q2W *every 2 weeks,* Q3W *every 3 weeks

### Impact of considered dose reductions on target engagement

To evaluate potential loss of pharmacological activity with 50% dose reductions, we used popPK-derived *C*_trough_ distribution to assess the fraction of patients who maintained serum *C*_trough_ levels that are associated with PD-L1 target occupancy (≥ 11 ug/mL) and with TGF-β neutralization (≥ 50 μg/mL), based on previously reported phase 1 PK-PD data [5]. Note that 50 μg/mL target *C*_trough_ for selections of RP2D was determined based on complete inhibition of all four targets (PD-L1 target occupancy and TGF-β1, -2, and -3 neutralization in circulation) in most (> 95% at 1200 mg Q2W) patients, with TGF-β2 inhibition associated with *C*_trough_ ≥ 50 μg/mL and PD-L1, TGF-β1, and TGF-β3 inhibition associated with C_trough_ ≥ 11 μg/mL (Table 3). These data suggested that 50% dose reductions (i.e., dosing with 1200 mg Q3W and 600 mg Q2W doses) maintain PD-L1 inhibition by BA, while losing some of TGF-β2 inhibition. For example, with a single 1200 mg Q3W dose, ≈30% of patients are projected to have TGF-β2 inhibition at the end of the dosing interval, with TGF-β2 inhibition maintained in ≈87% of patients for 2 weeks of the 3-week cycle. Thus, the 50% BA dose reduction could be viewed as an intermittent TGF-β2 inhibition.

**Table 3:** Percentage of participants with various target C_trough_

	*C*_trough_,sd ≥ 50 μg/mL, %	*C*_trough_,sd ≥ 11 μg/mL, %	*C*_trough_,ss ≥ 50 μg/mL, %	*C*_trough_,ss ≥ 11 μg/mL, %
1200 mg Q2W	87.2	100	95.1	100
600 mg Q2W	20.8	100	60.2	100
2400 mg Q3W	82.3	99.9	87.8	100
1200 mg Q3W	30.4	98.7	50.5	98.9

*C*_trough_ trough concentration, *Q2W* every 2 weeks, *Q3W* every 3 weeks, *sd* single dose, *ss* steady state

### Integration of exposure-safety modeling and available PK-PD data with safety monitoring for management of bleeding AE

**Fig. 3:**
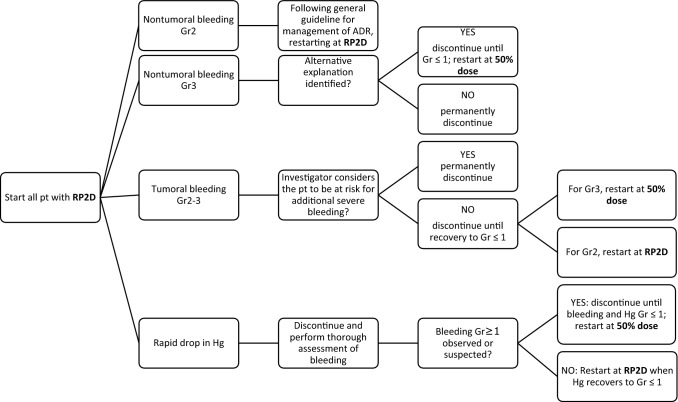
Guidance for management of bleeding adverse events in monotherapy and combination studies of bintrafusp alfa. Toxicity grading is assigned based on National Cancer Institute Common Terminology Criteria for Adverse Events version 5.0. Rapid drop in Hg is defined as decrease > 3 g/dL in 3 weeks or 2.0 g/dL in 2 weeks. Alternative explanations for nontumoral bleeding include concomitant use of antithrombotic agents, traumatic event. Thorough assessment of bleeding includes tests such as lower and upper GI endoscopy, enhancement CT. The guidance presented applies regardless of causality with the study intervention. General grade 2 ADR guideline: if a grade 2 ADR resolves to grade ≤ 1 by the last day of the current cycle, treatment may continue; if a grade 2 ADR does not resolve to grade ≤ 1 by the last day of the current cycle but is manageable and/or not clinically relevant, the medical monitor should be consulted to assess if it is clinically reasonable to administer the following infusion (at RP2D). *ADR* adverse drug reaction, *CT* computed tomography, *Gr* grade, *Hg* hemoglobin, *pt* participant, *Gr ≤ 1* resolved or Gr 1

Based on available exposure-safety modeling results, PK-PD data, and clinical considerations, the prophylactic dose modification approach was implemented in ongoing monotherapy and combination studies, as shown in Fig. 3. The guidance on whether and when to temporarily discontinue treatment, permanently discontinue treatment, restart at RP2D or restart at 50% dose depends on type of bleeding (tumoral versus nontumoral), the grade of bleeding AE, and investigator assessment of risk of additional bleeding. It is noted that further dose increase from 50% of RP2D to full RP2D was allowed in study protocols, following patient stabilization and consultation with the medical monitor.

We note that the proposal is based on the assumption that the exposure-safety relationship observed with the initial dose is relevant to the probability of recurring episodes of bleeding events.

## Discussion

Here we report a pragmatic approach for prevention of recurrence and management of bleeding AEs for a bifunctional therapeutic protein BA. Specifically, the proposed approach for management of observed or suspected bleeding AEs of grade 3 is temporary treatment discontinuation, followed by a restart of the treatment with 50% of the initial dose upon the resolution of the initial bleeding event. This approach was informed by exposure-safety modeling and PK-PD understanding for target engagement by BA and the current understanding of the bifunctional mode of action for both efficacy and safety. To our knowledge, this is the first example of a dose modification for AE management of a therapeutic protein other than antibody–drug conjugate. Dose modification strategies were reported for some antibody–drug conjugates primarily to mitigate toxicities associated with the small molecule cytotoxic payload [25].

It is noted that the model-derived numerical estimates for the probability of bleeding should be interpreted with caution and there are no data available on probability of recurrence of bleeding AE upon re-initiation of treatment. We acknowledge that the current analysis does not address estimation of exposure–AE relationships in individual patients, as the protocol recommended to withhold or discontinue treatment at the time of analysis and did not allow for dose modifications after resolution of AEs. Accordingly, separating within- and between-patient sources of variability in exposure-safety relationships is not feasible with the available data. Dose reductions for treatment-emergent toxicities were not specified in this study since the typical AE management approach for a therapeutic protein is treatment interruption or discontinuation upon first occurrence of an AE. Therefore, longitudinal data on recurrent AEs with dose modifications are not available to date. Nevertheless, with a substantial dataset (*n* = 936) and the range of systemic exposures observed in the dataset owing to pharmacokinetic variability, a robust exposure-safety relationship for the first bleeding AE could be described. This indicates exposure-relatedness of the observed AE, thereby supporting the conclusion from this analysis that implementation of a dose-reduction scheme should be expected to mitigate treatment-emergent toxicities. We posit, based on these results, that dose reduction without permanent treatment discontinuation would be preferable for managing bleeding toxicities so that the patient can have a chance to benefit from the drug. Thus, these analyses are considered “fit for purpose” for informing whether a dose reduction to ≈50% of the original dose for management of bleeding AEs is a reasonable approach given the current understanding of exposure-safety and PK-PD considerations for target engagement. The 50% dose reduction from RP2D (i.e., from 2400 mg Q3W to 1200 mg Q3W or from 1200 mg Q2W to 600 mg Q2W) was chosen based on the integration of all available information, such as (1) clinically meaningful reduction of probability of GI bleed, as estimated from exposure-safety model; (2) expected reduction in TGF-β trapping at the end of dosing interval (especially TGF-β2), with maintained PD-L1 inhibition, aimed at improving benefit-risk profile for patients at higher risk of bleeding (based on prior occurrence); and (3) ease of implementation, given that the 1200 mg dose is already in use in clinical trials and the clinical vial size is 600 mg.

While TGF-β inhibition has been reported to be associated with an increased risk of bleeding, the relative contribution of the three TGF-β isoforms to bleeding AE is not known. However, it is hypothesized that TGF-β2 may be important for at least some of the toxicities associated with TGF-β inhibition including bleeding, since TGF-β2 is thought to be a positive regulator of hematopoiesis and normal cardiac function [26, 27]. In fact, there are several anti-TGF-β agents in preclinical development that target TFG-β1 and -3 but not TFG-β2 [28, 29]. In line with the low intrinsic binding affinity between TGF-β2 and TGF-β receptor II [4], the phase 1 PK-PD profile indicated that BA is more selective in blocking TGF-β1 and -3 compared with TGF-β2 [5]. These data supported the notion that a dose reduction approach for restarting the treatment may reduce the probability of bleeding AEs while retaining pharmacological activity of PD-L1 axis and some activity for TGF-β1 and -3 inhibition in most patients.

The proposed BA dose modification for mitigation of risk of recurring bleeding AEs is expected to result in the intermittent TGF-β2 inhibition upon re-initiation of dosing in ≈50% to 70% of the patients. Interestingly, for galunisertib, a small molecule TGF-β pathway inhibitor, an intermittent dosing schedule, informed by preclinical PK-PD modeling, was employed to mitigate the risk of TGF-β-associated cardiac toxicity observed in animal toxicology studies [19].

Evaluation of sensitivity to ethnic factors is an important component of global oncology therapeutic development to enable Asia-inclusive development strategies applying International Council for Harmonisation E5 and E17 principles and Totality of Evidence concepts [31–34]. The present analysis was based on a comprehensive dataset including both Asian (31.5%) and non-Asian (68.5%) patients represented across the global clinical development program for BA. Covariate analyses in the exposure-safety logistic regression models did not identify Asian race as a predictor of bleeding AEs. Taken together with the results of previously reported population PK analyses of BA that concluded consistent systemic exposures in Asian and non-Asian populations [35], these results support continued evaluation of a common approach to bleeding risk management across global populations in Asia-inclusive clinical development of BA.

Finally, it is noted that there were insufficient data to evaluate the contribution of chemotherapy to the probability of bleeding when coadministered with BA, since all patients in the chemotherapy combination cohorts in NCT03840915 were treated with 2400 mg Q3W, a relatively high dose. However, as demonstrated by the sensitivity analysis (Supplemental Fig. 1), a similar exposure–response relationship for bleed_TEAE and bleed_GI was observed with monotherapy-only data compared with monotherapy plus chemotherapy combination data. Furthermore, after examining the data from NCT03840915 alone, which by itself was not sufficient for independent exposure-safety modeling, an exposure-related increase in the incidence of bleeding AEs was observed. Taken together, these observations support confidence in the use of the developed exposure-safety model to inform design of dose modification guidelines to manage treatment-emergent bleeding, an approach that has now been implemented across the BA global clinical development program.

In summary, we have described a pragmatic approach for prevention and management of bleeding AEs for a bifunctional therapeutic protein BA, which has been implemented in ongoing phase 1 to 3 clinical trials. While bleeding AEs of grade ≥ 3 are relatively rare (most bleeding observed was mild to moderate) and typically do not lead to treatment discontinuation, the incidence of bleeding was higher than that for a typical immunotherapy agent and warranted additional prophylactic risk mitigation measures. Exposure-safety and PK-PD evaluations support the proposed 50% dose reduction for restarting the treatment after the treatment interruption and resolution of the initial bleeding event. This dose modification approach is expected to improve the benefit-risk profile in the patients by retaining pharmacological activity of the PD-L1 axis of BA and maintaining patients on treatment, while decreasing the recurrence of bleeding AEs. The approach for management of bleeding AEs is consistent with the current understanding of the bifunctional nature of the protein and contribution of each target for efficacy and safety. Further investigations are needed to evaluate effectiveness of this approach (including longitudinal modeling), based on safety data generated after its implementation. In addition, longitudinal modeling approaches (including those accounting for the immortal time bias [36]) may be considered to evaluate the exposure-safety relationship for re-occurrence of bleeding AEs. This example highlights that pragmatic dosing modification strategies may be considered to improve the benefit-risk profile for therapeutic proteins. Exposure-safety and PK-PD modeling and simulations coupled with understanding of mechanism of AEs of special interest are critical to inform these strategies.

## Supplementary Information

Below is the link to the electronic supplementary material.

Supplementary file1 (PDF 704 KB)

## References

[1] Lind H, Gameiro SR, Jochems C, Donahue RN, Strauss J, Gulley JM, Palena C, Schlom J (2020). Dual targeting of TGF-beta and PD-L1 via a bifunctional anti-PD-L1/TGF-betaRII agent: status of preclinical and clinical advances. J Immunother Cancer.

[2] Wrzesinski SH, Wan YY, Flavell RA (2007). Transforming growth factor-beta and the immune response: implications for anticancer therapy. Clin Cancer Res.

[3] Tzavlaki K, Moustakas A (2020). TGF-beta signaling. Biomolecules.

[4] Lin HY, Moustakas A, Knaus P, Wells RG, Henis YI, Lodish HF (1995). The soluble exoplasmic domain of the type II transforming growth factor (TGF)-beta receptor. A heterogeneously glycosylated protein with high affinity and selectivity for TGF-beta ligands. J Biol Chem.

[5] Vugmeyster Y, Wilkins J, Koenig A, El Bawab S, Dussault I, Ojalvo LS, De Banerjee S, Klopp-Schulze L, Khandelwal A (2020). Selection of the recommended phase 2 dose for bintrafusp alfa, a bifunctional fusion protein targeting TGF-beta and PD-L1. Clin Pharmacol Ther.

[6] Vugmeyster Y, Klopp-Schulze L, Rueckert P, Khandelwal A, Speit I, Osada M, Grenga I (2020) Safety and pharmacokinetics of bintrafusp alfa with Q3W dosing: confirmation of the model-informed dose selection. ACOP 2020 Virtual Conference; November 9–13, 2020

[7] Paz-Ares L, Kim TM, Vicente D, Felip E, Lee DH, Lee KH, Lin CC, Flor MJ, Di Nicola M, Alvarez RM, Dussault I, Helwig C, Ojalvo LS, Gulley JL, Cho BC (2020). Bintrafusp alfa, a bifunctional fusion protein targeting TGF-beta and PD-L1, in second-line treatment of patients with NSCLC: results from an expansion cohort of a phase 1 trial. J Thorac Oncol.

[8] Strauss J, Braiteh FS, Calvo E, Miguel MD, Cervantes A, Edenfield WJ, Li T, Rasschaert MA, Park-Simon T-W, Longo F, Paz-Ares LG, Spira AI, Jehl G, Dussault I, Ojalvo LS, Gulley JL, Allan SW (2021) Evaluation of bintrafusp alfa, a bifunctional fusion protein targeting TGF-β and PD-L1, in cervical cancer: data from phase 1 and phase 2 studies. J Clin Oncol 39 (15_suppl):5509. 10.1200/JCO.2021.39.15_suppl.5509

[9] Strauss J, Heery CR, Schlom J, Madan RA, Cao L, Kang Z, Lamping E, Marte JL, Donahue RN, Grenga I, Cordes L, Christensen O, Mahnke L, Helwig C, Gulley JL (2018). Phase I trial of M7824 (MSB0011359C), a bifunctional fusion protein targeting PD-L1 and TGFβ, in advanced solid tumors. Clin Cancer Res.

[10] Yoo C, Oh DY, Choi HJ, Kudo M, Ueno M, Kondo S, Chen LT, Osada M, Helwig C, Dussault I, Ikeda M (2020). Phase I study of bintrafusp alfa, a bifunctional fusion protein targeting TGF-beta and PD-L1, in patients with pretreated biliary tract cancer. J Immunother Cancer.

[11] Doi T, Fujiwara Y, Koyama T, Ikeda M, Helwig C, Watanabe M, Vugmeyster Y, Kudo M (2020) Phase I study of the bifunctional fusion protein bintrafusp alfa in Asian patients with advanced solid tumors, including a hepatocellular carcinoma safety-assessment cohort. Oncologist 25:e1292-e1302. 10.1634/theoncologist.2020-0249 10.1634/theoncologist.2020-0249 PMC7485354 32324927

[12] Rolfo C, Greillier L, Veillon; R, Badin F, Ghiringhelli F, Isambert N, Paulus A, Lambrechts M, Chaudhary S, Xiaoli You X, Vugmeyster Y, Helwig C, Hiret S (2021) Bintrafusp alfa in combination with chemotherapy in patients with stage IV NSCLC: safety and pharmacokinetic results of the INTR@PID LUNG 024 study, Poster 465. Paper presented at the Society for Immunotherapy fo Cancers 37th Annual Meeting, Washington, DC; November 10–14, 2021

[13] Gulley JL, Lacouture ME, Spira A, Verdaguer Mata H, Yoo C, Cho BC, Helwig C, Halady T, Valencia C, Bajars M, Strauss J, Brownell I (2021). 1689P Adverse event management during treatment with bintrafusp alfa, a bifunctional fusion protein targeting TGF-β and PD-L1: treatment guidelines based on experience in clinical trials. Ann Oncol.

[14] Morris JC, Tan AR, Olencki TE, Shapiro GI, Dezube BJ, Reiss M, Hsu FJ, Berzofsky JA, Lawrence DP (2014). Phase I study of GC1008 (fresolimumab): a human anti-transforming growth factor-beta (TGFbeta) monoclonal antibody in patients with advanced malignant melanoma or renal cell carcinoma. PLoS ONE.

[15] Kim BG, Malek E, Choi SH, Ignatz-Hoover JJ, Driscoll JJ (2021). Novel therapies emerging in oncology to target the TGF-beta pathway. J Hematol Oncol.

[16] Martins F, Sofiya L, Sykiotis GP, Lamine F, Maillard M, Fraga M, Shabafrouz K, Ribi C, Cairoli A, Guex-Crosier Y, Kuntzer T, Michielin O, Peters S, Coukos G, Spertini F, Thompson JA, Obeid M (2019). Adverse effects of immune-checkpoint inhibitors: epidemiology, management and surveillance. Nat Rev Clin Oncol.

[17] Wang PF, Chen Y, Song SY, Wang TJ, Ji WJ, Li SW, Liu N, Yan CX (2017). Immune-related adverse events associated with anti-PD-1/PD-L1 treatment for malignancies: a meta-analysis. Front Pharmacol.

[18] Mitra MS, Lancaster K, Adedeji AO, Palanisamy GS, Dave RA, Zhong F, Holdren MS, Turley SJ, Liang WC, Wu Y, Meng YG, Vernes JM, Schutten MM (2020). A potent pan-TGFβ neutralizing monoclonal antibody elicits cardiovascular toxicity in mice and cynomolgus monkeys. Toxicol Sci.

[19] Herbertz S, Sawyer JS, Stauber AJ, Gueorguieva I, Driscoll KE, Estrem ST, Cleverly AL, Desaiah D, Guba SC, Benhadji KA, Slapak CA, Lahn MM (2015). Clinical development of galunisertib (LY2157299 monohydrate), a small molecule inhibitor of transforming growth factor-beta signaling pathway. Drug Des Devel Ther.

[20] Kewan T, Covut F, Ahmed R, Haddad A, Daw H (2020). Clinically significant bleeding with immune checkpoint inhibitors: a retrospective cohort study. Eur J Cancer.

[21] Leighl NB, Bennouna J, Yi J, Moore N, Hambleton J, Hurwitz H (2011). Bleeding events in bevacizumab-treated cancer patients who received full-dose anticoagulation and remained on study. Br J Cancer.

[22] Wang Y, Booth B, Rahman A, Kim G, Huang SM, Zineh I (2017). Toward greater insights on pharmacokinetics and exposure-response relationships for therapeutic biologics in oncology drug development. Clin Pharmacol Ther.

[23] Dai HI, Vugmeyster Y, Mangal N (2020). Characterizing exposure-response relationship for therapeutic monoclonal antibodies in immuno-oncology and beyond: challenges, perspectives, and prospects. Clin Pharmacol Ther.

[24] ICH (2020) Introductory Guide for Standardised MedDRA Queries (SMQs) Version 23.0. https://admin.meddra.org/sites/default/files/guidance/file/SMQ_intguide_23_0_English.pdf. Accessed November 2021

[25] Donaghy H (2016). Effects of antibody, drug and linker on the preclinical and clinical toxicities of antibody-drug conjugates. MAbs.

[26] Bartram U, Molin DG, Wisse LJ, Mohamad A, Sanford LP, Doetschman T, Speer CP, Poelmann RE, Gittenberger-de Groot AC (2001). Double-outlet right ventricle and overriding tricuspid valve reflect disturbances of looping, myocardialization, endocardial cushion differentiation, and apoptosis in TGF-beta(2)-knockout mice. Circulation.

[27] Langer JC, Henckaerts E, Orenstein J, Snoeck HW (2004). Quantitative trait analysis reveals transforming growth factor-beta2 as a positive regulator of early hematopoietic progenitor and stem cell function. J Exp Med.

[28] Varricchio L, Iancu-Rubin C, Upadhyaya B, Zingariello M, Martelli F, Verachi P, Clementelli C, Denis JF, Rahman AH, Tremblay G, Mascarenhas J, Mesa RA, O'Connor-McCourt M, Migliaccio AR, Hoffman R (2021). TGF-beta1 protein trap AVID200 beneficially affects hematopoiesis and bone marrow fibrosis in myelofibrosis. JCI Insight.

[29] Martin CJ, Datta A, Littlefield C, Kalra A, Chapron C, Wawersik S, Dagbay KB, Brueckner CT, Nikiforov A, Danehy FT Jr, Streich FC Jr, Boston C, Simpson A, Jackson JW, Lin S, Danek N, Faucette RR, Raman P, Capili AD, Buckler A, Carven GJ, Schürpf T (2020) Selective inhibition of TGFbeta1 activation overcomes primary resistance to checkpoint blockade therapy by altering tumor immune landscape. Sci Transl Med 12:eaay8456. 10.1126/scitranslmed.aay8456 10.1126/scitranslmed.aay8456 32213632

[30] Boilève A, Hilmi M, Smolenschi C, Ducreux M, Hollebecque A, Malka D (2021). Immunotherapy in advanced biliary tract cancers. Cancers (Basel).

[31] Li C, Wang B, Lu D, Jin JY, Gao Y, Matsunaga K, Igawa Y, Nijem I, Lu M, Strasak A, Chernyukhin N, Girish S (2016). Ethnic sensitivity assessment of the antibody-drug conjugate trastuzumab emtansine (T-DM1) in patients with HER2-positive locally advanced or metastatic breast cancer. Cancer Chemother Pharmacol.

[32] Venkatakrishnan K, Burgess C, Gupta N, Suri A, Takubo T, Zhou X, DeMuria D, Lehnert M, Takeyama K, Singhvi S, Milton A (2016). Toward optimum benefit-risk and reduced access lag for cancer drugs in Asia: a global development framework guided by clinical pharmacology principles. Clin Transl Sci.

[33] Venkatakrishnan K, Cook J (2018). Driving access to medicines with a totality of evidence mindset: an opportunity for clinical pharmacology. Clin Pharmacol Ther.

[34] Walsh R, Goh BC (2019). Population diversity in oncology drug responses and implications to drug development. Chin Clin Oncol.

[35] Wilkins JJ, Vugmeyster Y, Dussault I, Girard P, Khandelwal A (2019). Population pharmacokinetic analysis of bintrafusp alfa in different cancer types. Adv Ther.

[36] Khandelwal A, Grisic AM, French J, Venkatakrishnan K (2022). Pharmacometrics golems: exposure-response models in oncology. Clin Pharmacol Ther.

