# Supplementary material for: Model-informed approach for risk management of bleeding toxicities for bintrafusp alfa, a bifunctional fusion protein targeting TGF-β and PD-L1
Source: Cancer Chemother Pharmacol. 2022 Sep 6;90(4):369–79. doi: 10.1007/s00280-022-04468-6 (PMC9474582; doi:10.1007/s00280-022-04468-6)

SUPPLEMENTAL MATERIAL

Supplemental Methods

Population pharmacokinetic model development

The software packages NONMEM (version 7.3.0), Perl-speaks-NONMEM (PsN; http://psn.sourceforge.net/index.php, version 4.4.8), and Pirana (https://www.certara.com/software/pirana-modeling-workbench/, version 2.9.2) were used for model development in this analysis, while pre- and post-processing were performed using R (http://www.r-project.org/ version 3.5.1) and RStudio (version 1.0.153). The estimation method used for nonlinear mixed-effects model development was first-order conditional with interaction. Prior to model development, an exploratory analysis was performed (e.g., to investigate relationships between observed drug concentration and time or covariates of interest). Precision of the parameter estimates was assessed via the covariance matrix, derived from the Fisher information matrix obtained in the covariance step. Stability of the models was assessed on the basis of convergence criteria, goodness-of-fit plots, successful covariance steps, condition number (<1000), and correlation between parameters. Model selection was based on the difference in the objective function value (OFV) between 2 nested models for hierarchical models ($p<0.05$), decrease in unexplained variability, improvement in goodness-of-fit plots, and scientific and (patho)physiological plausibility of the model. To evaluate the predictive performance of the models, prediction-corrected visual predictive checks (pcVPCs) were performed. Between-subject variability in CL and V_1 was assumed to be log-normally distributed (exponential), and for maximal change in CL over time (I_{max}) it was assumed to be normally distributed (additive). Residual unexplained variability was described using a combined additive and proportional error model. Covariate analysis included the following steps: (1) refinement of pre-defined list of covariates of interest, including assessment of correlation among covariates and proportion of missing covariate values; (2) inclusion of the covariates from the refined list on CL and V_1 simultaneously, resulting in the full model; (3) full model reduction, including backwards elimination via SCM functionality in PsN.

Supplemental Tables

Supplementary Table 1. Parameter estimates from the final population PK model

Parameter, unit	***Estimate (RSE) [shrinkage]***
Clearance CL, L/h	0.0167 (2%)
Central volume of distribution V_1, L	3.33 (1%)
Peripheral volume of distribution V_2, L	0.356 (14%)
Intercompartmental exchange rate Q, L/h	0.00423 (37%)
Time to half-maximal change in CL over time, T_{50}, days	32.8 (10%)
Curve shape factor γ	3.40 (42%)
Maximum change in time-varying CL I_{max}	−0.0296 (52%)
Effect of body weight on CL and Q	0.448 (13%)
Effect of body weight on V_1 and V_2	0.364 (13%)
Effect of ADA on CL	0.0472 (35%)
Effect of albumin on CL	−0.400 (21%)
Effect of CRP on CL	0.000853 (47%)
Effect of eGFR on CL	0.0928 (28%)
Effect of INR on CL	0.230 (20%)
Effect of platelets on CL	0.0626 (53%)
Effect of sex on CL	−0.118 (13%)
Effect of tumor size on CL	0.0546 (25%)
Effect of tumor type: NSCLC PDL1 fail on CL	−0.0741 (27%)
Effect of tumor type: pancreatic/HCC on CL	−0.0820 (24%)
Effect of tumor type: NSCLC on CL	−0.0787 (27%)
Effect of tumor type: Esoph.SCC/Cervical on CL	−0.0853 (30%)
Effect of tumor type: glioblastoma on CL	−0.227 (11%)
Effect of tumor type: SCCHN on CL	−0.102 (25%)
Effect of WBC on CL	0.0759 (47%)
Effect of albumin on V_1	−0.195 (35%)
Effect of INR on V_1	0.148 (30%)
Effect of platelets on V_1	0.0398 (67%)
Effect of sex on V_1	−0.133 (9%)

Effect of tumor size on V_1	0.0238 (41%)
Effect of WBC on V_1	−0.0927 (28%)
Between-subject variability in CL, CV%	19.6 (4%) [10%]
Between-subject variability in V_1, CV%	14.8 (4%) [14%]
Between-subject variability in I_{max}, CV%	18.9 (13%) [32%]
Proportional RUV, CV%	0.183 (2%)
Additive RUV, μg/mL	2.62 (31%)

ADA, anti-drug antibodies; CRP, C-reactive protein; eGFR, estimated glomerular filtration rate; Esoph.SCC, esophageal squamous cell carcinoma; HCC, hepatocellular carcinoma; INR, international normalized ratio; NSCLC, non-small cell lung cancer; PD-L1, programmed death ligand 1; PK, pharmacokinetic; RSE, relative standard error; RUV, residual unexplained variability; SCCHN, squamous cell carcinoma of the head and neck; WBC, white blood cell count.

Time-varying CL was parametrized as follows: $CL = CL_{TV} \cdot e^{(I_{max}+\eta_{Imax})\cdot t^{\gamma}/({t_{50}}^{\gamma}+t^{\gamma})} \cdot e^{\eta_{CL}}$

Covariates were parametrized as follows:

$$Effect\ of\ body\ weight = \left(\frac{BW}{70}\right)^{\theta_{BW}}$$

$$Effect\ of\ ADA\ ever\ positive = 1 + \theta_{ADA}$$

$$Effect\ of\ albumin = \left(\frac{albumin}{39.2}\right)^{\theta_{albumin}}$$

$$Effect\ of\ CRP = e^{\theta_{CRP}\cdot(CRP-8)}$$

$$Effect\ of\ eGFR = \left(\frac{eGFR}{93.29}\right)^{\theta_{eGFR}}$$

$$Effect\ of\ INR = \left(\frac{INR}{1.04}\right)^{\theta_{INR}}$$

$$Effect\ of\ platelet\ count = \left(\frac{PC}{222}\right)^{\theta_{PC}}$$

$$Effect\ of\ male\ sex = 1 + \theta_{sex}$$

$$Effect\ of\ tumor\ size = \left(\frac{TS}{63}\right)^{\theta_{TS}}$$

$$Effect\ of\ NSCLC\ PDL1\ fail\ tumor\ type = 1 + \theta_{TT1}$$

$$Effect\ of\ pancreatic/HCC\ tumor\ type = 1 + \theta_{TT2}$$

$$Effect\ of\ NSCLC\ tumor\ type = 1 + \theta_{TT3}$$

$$Effect\ of\ Esoph.SCC/cervical\ tumor\ type = 1 + \theta_{TT4}$$

$$Effect\ of\ glioblastoma\ tumor\ type = 1 + \theta_{TT5}$$

$$Effect\ of\ SCCHN\ tumor\ type = 1 + \theta_{TT6}$$

$$Effect\ of\ WBC = \left(\frac{WBC}{6.48}\right)^{\theta_{WBC}}$$

Supplemental Table 2. Dataset for multivariable exposure-safety analysis

Variable	NCT02517398	NCT02699515	NCT03840915	NCT04246489	Total
N	593	114	70	159	936
Bintrafusp alfa $C_{avg,SD}$, median (geometric mean [range], µg/mL	172 (173) [6.12-554]	177 (171) [20.2-303]	245 (244) [145-400]	177 (178) [111-268]	178 (179) [6.12-554]
Age, median (geometric mean [range], y	61 (60.6) [25-90]	62 (60.5) [38-82]	62 (62.2) [40-83]	65 (64) [39-83]	62 (61.3) [25-90]
Body weight, median (geometric mean [range], kg	71.7 (74) [40.6-128]	57.6 (57.8) [33.5-97.1]	77 (78.7) [38.1-151]	65 (66.8) [38-101]	68.5 (71.2) [33.5-151]
Sex, n (%)					
Male	381 (64.2)	82 (71.9)	47 (67.1)	94 (59.1)	604 (64.5)
Female	212 (35.8)	32 (28.1)	23 (32.9)	65 (40.9)	332 (35.5)
Race, n (%)					
Asian	104 (17.5)	114 (100)	–	77 (48.4)	295 (31.5)
Non-Asian	489 (82.5)	–	70 (100)	82 (51.6)	641 (68.5)
Renal impairment, n (%)					
Normal	324 (54.6)	79 (69.3)	41 (58.6)	81 (50.9)	525 (56.1)
Mild	232 (39.1)	32 (28.1)	26 (37.1)	55 (34.6)	345 (36.9)
Moderate	37 (6.24)	3 (2.63)	3 (4.29)	23 (14.5)	66 (7.05)
Hepatic impairment, n (%)					
Normal	456 (76.9)	89 (78.1)	62 (88.6)	102 (64.2)	709 (75.7)
Mild	132 (22.3)	25 (21.9)	7 (10)	55 (34.6)	219 (23.4)
Moderate	5 (0.843)	–	–	2 (1.26)	7 (0.748)
Severe	–	–	1 (1.43)	–	1 (0.107)
Baseline albumin, median (geometric mean [range], g/L	39 (38.9) [21-49.2]	40 (39.3) [25-48]	38.6 (38) [26-47]	39.2 (39.5) [30-49.2]	39 (39) [21; 49.2]
International normalized ratio median (geometric mean [range]	1.04 (1.1) [0.8-4.2]	1.04 (1.06) [0.89-2.06]	1 (1.08) [0.8-3.3] (5)	1.03 (1.08) [0.89-2.07]	1.03 (1.09) [0.8-4.2] (5)
Baseline neutrophil-lymphocyte ratio median (geometric mean [range]	3.88 (5) [0.02-95.9]	3.26 (3.78) [0.57-22.2]	6.56 (8.29) [1.57-40.1]	3.03 (3.95) [0.79-20.7]	3.75 (4.92) [0.02-95.9]
Baseline white blood cell count, median (geometric mean [range], 10^9/L	6.6 (7.14) [2.4-33.2]	5.91 (6.58) [3.1-26.6]	9.41 (9.77) [4.9-27.3]	6.4 (7.3) [3.3-27]	6.6 (7.29) [2.4-33.2]
Baseline platelet count, median (geometric mean [range], 10^9/L	231 (254) [58; 740]	219 (245) [66; 837]	293 (317) [146; 652]	183 (203) [75; 606]	227 (249) [58; 837]

Variable	NCT02517398	NCT02699515	NCT03840915	NCT04246489	Total
Baseline tumor size, median (geometric mean [range], mm	70 (82.4) [10; 370] (36)	51 (62.2) [11; 275]	91 (103) [14; 281] (1)	68 (73.6) [10; 231]	67 (79.8) [10; 370] (37)
Tumor type, n (%)					
BTC	–	30 (26.3)	–	159 (100)	189 (20.2)
Cervical	23 (3.88)	–	–	–	23 (2.46)
NSCLC	204 (34.4)	–	70 (100)	–	274 (29.3)
Other	366 (61.7)	84 (73.7)	–	–	450 (48.1)
Baseline ECOG score, n (%)					
0	208 (35.1)	42 (36.8)	21 (30)	92 (57.9)	363 (38.8)
≥1	385 (64.9)	72 (63.2)	49 (70)	67 (42.1)	573 (61.2)
Concomitant corticosteroids, n (%)					
No	325 (54.8)	64 (56.1)	–	107 (67.3)	496 (53)
Yes	268 (45.2)	50 (43.9)	70 (100)	52 (32.7)	440 (47)
Concomitant antibiotics, n (%)					
No	574 (96.8)	114 (100)	46 (65.7)	159 (100)	893 (95.4)
Yes	19 (3.2)	–	24 (34.3)	–	43 (4.59)

Continuous variables are expressed as median (geometric mean) [range] (N missing/lower limit of quantitation). Categorical variables are expressed as n (%). Data cutoffs were May 15, 2020, for NCT02517398 and NCT02699515, September 30, 2020, for NCT04246489, and November 25, 2020, for NCT03840915.

Supplemental Table 3. Final exposure-safety models for bleed_TEAE and bleed_GI

Parameter	Adverse event	
	**Bleed_TEAE**	**Bleed_GI**
Bintrafusp alfa $C_{avg,SD}$ (per 100 ug/mL)	1.78 (1.37-2.32)	1.87 (1.34-2.62)
Baseline age (per 10 y)		0.789 (0.665-0.936)
Sex: female	0.672 (0.489-0.919)	0.661 (0.436-0.989)
Hepatic impairment		1.52 (1.01-2.27)
Renal impairment		1.68 (1.15-2.46)
Tumor type: BTC	0.345 (0.228-0.51)	
Tumor type: Cervical	3.92 (1.59-10.6)	

Numbers are odds ratios (95% CIs).
bleed_GI, gastrointestinal bleeding event of any grade; bleed_TEAE, treatment-related bleeding event of any grade; BTC, biliary track cancer; $C_{avg,SD}$, average concentration over the dosing interval
Note: data were analyzed by logistic regression, using the following equation:

$$\text{logit}(p) = \log\left(\frac{p(AE = 1)}{1 - p(AE = 1)}\right) = \beta_0 + \beta_1 \cdot x_1 + \beta_2 \cdot x_2 + \ldots + \beta_n \cdot x_n$$

where p is the probability of adverse event AE, B_0 is the probability of AE absent any predictors, $x_{1\ldots n}$ is a vector of n predictors, and $\beta_{1\ldots n}$ is a vector of coefficients describing the effects of $x_{1\ldots n}$ on p.

Supplemental Figures

Supplemental Figure 1. Goodness-of-fit plots for the final population pharmacokinetic model.

Purple points are individual data points; yellow lines are locally weighted smoothing curves. CWRESI, conditional weighted residuals.

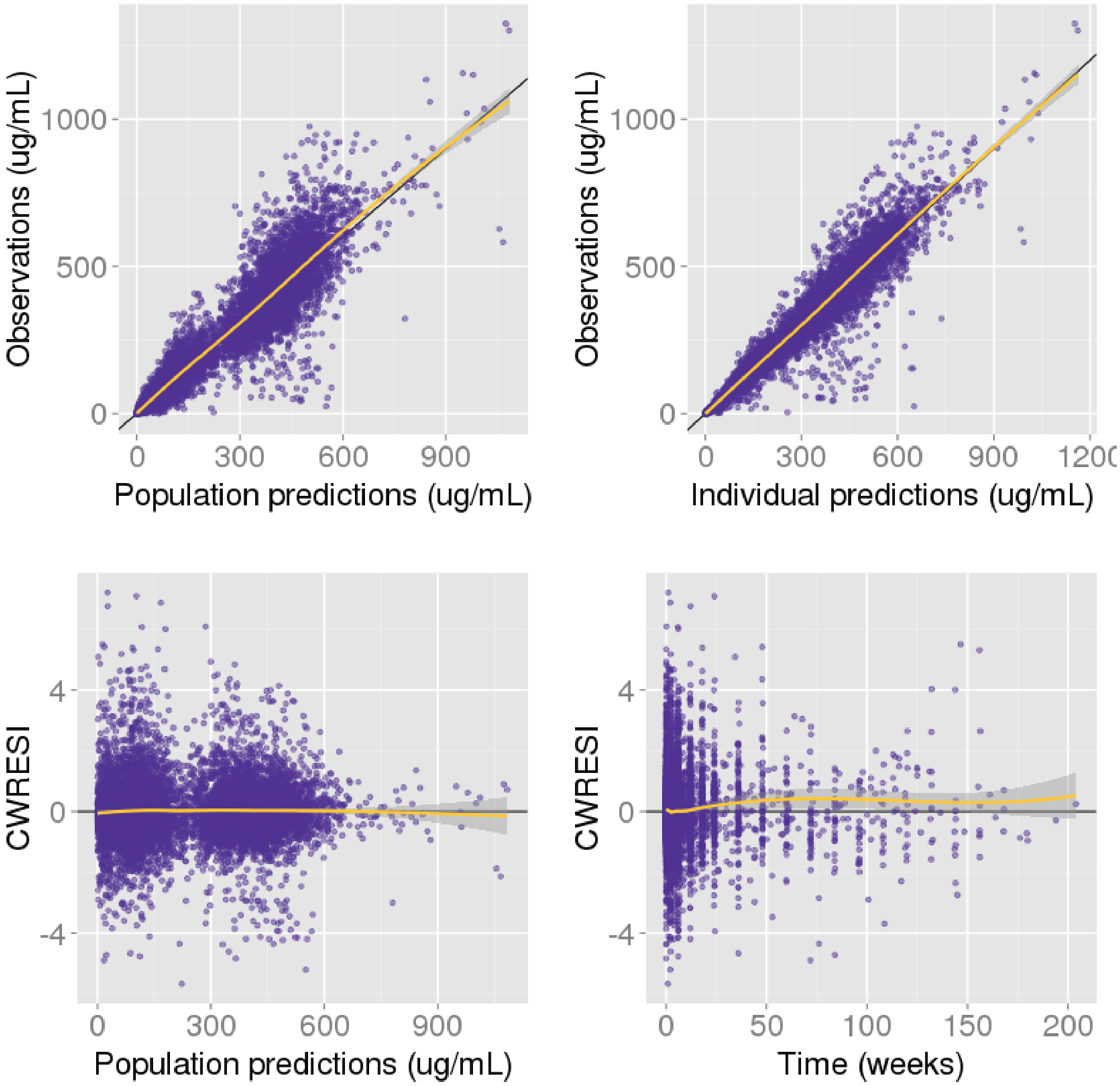

Supplemental Figure 2. Prediction-corrected visual predictive checks for the full pharmacokinetic model for bintrafusp alfa.

Points are individual observations; lines represent medians, 5th and 95th percentiles of the binned observed data; and shaded areas represent 95% prediction intervals around the median and the 5th and 95th percentiles based on the simulations. The X-axis shows time since previous dose for every observation.

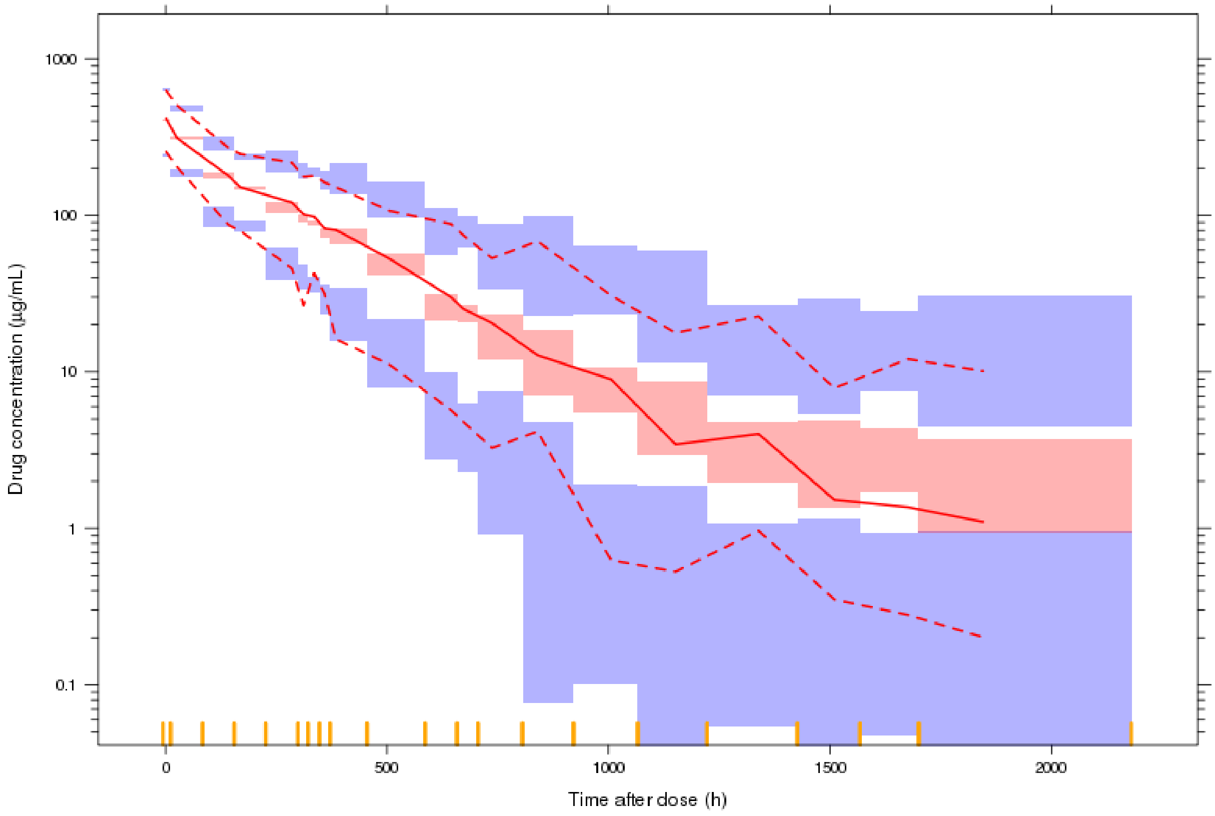

Supplemental Figure 3. Univariable exposure-safety analysis for bleed_TEAE (A) and bleed_GI (B) based on monotherapy studies NCT02517398, NCT02699515, and NCT04246489.

The blue line and shaded area represent model-predicted AE probability (median and 95% CI); pink circles represent observed AE incidence by quartiles of exposure and are placed at the 12.5th, 37.5th, 62.5th, and 87.5th percentiles of the exposure distribution (i.e. the median for each exposure quartile); the error bars represent 95% CIs; pink dotted lines represent boundaries of exposure quartiles; and purple dots indicate individual patient data. The odds ratio was 1.59 (95% CI 1.22-2.11) per 100 µg/mL for Bleed_TEAE and 1.69 (95% CI 1.2-2.42) per 100 µg/mL for Bleed_GI. AE, adverse event; $C_{avg,SD}$, average bintrafusp alfa concentration over the dosing interval; bleed_TEAE, treatment-related bleeding event of any grade; bleed_GI, gastrointestinal bleeding events of any grade.

A. Bleed_TEAE

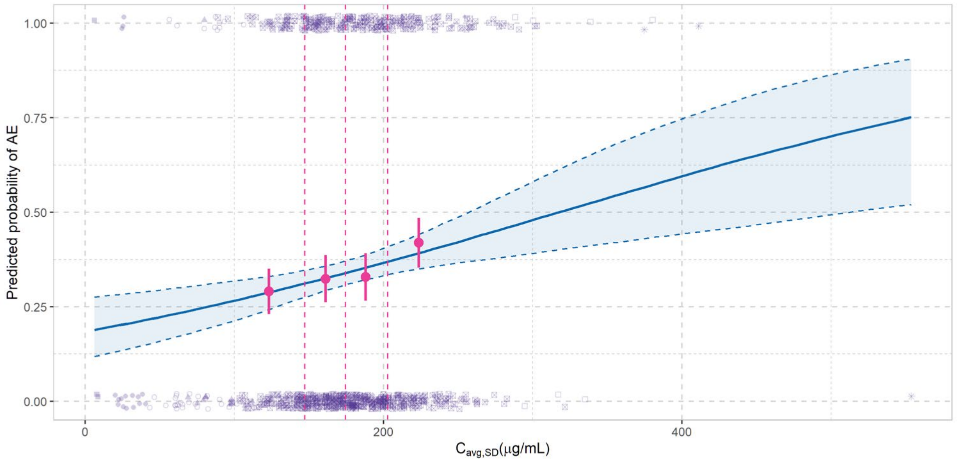

B. Bleed_GI

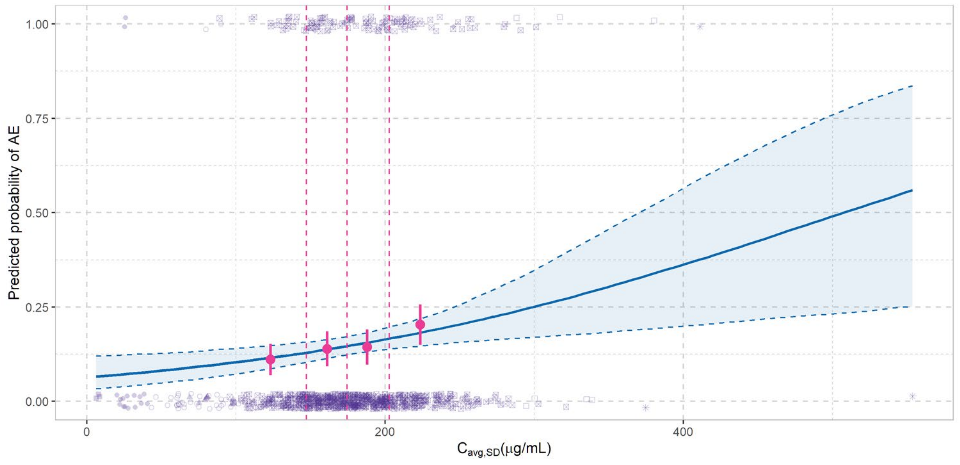

Supplement: Supplementary file 1 — Supplementary file1 (PDF 704 KB) [file 280_2022_4468_MOESM1_ESM.pdf]
